# Supplementary material for: Hydrothermal Synthesis of Functionalized Carbon Nanodots and Their Clusters as Ionic Probe for High Sensitivity and Selectivity for Sulfate Anions with Excellent Detection Level
Source: Polymers (Basel). 2023 Jun 12;15(12):2655. doi: 10.3390/polym15122655 (PMC10302774; doi:10.3390/polym15122655)
Supplement: Supplementary file 1 [file polymers-15-02655-s001.zip › polymers-2439088-supplementary.pdf]

polymers

Supplementary Material

Hydrothermal Synthesis of Functionalized Carbon Nanodots and Their Clusters as Ionic Probe for High Sensitivity and Selectivity for Sulfate Anions with Excellent Detection Level

Po-Chih Yang [1,*], Pradeep Kumar Panda [1], Cheng-Han Li [1], Yu-Xuan Ting [1], Yasser Ashraf Gandomi [2] and Chien-Te Hsieh [1,*]

[1] Department of Chemical Engineering and Materials Science, Yuan Ze University, Taoyuan 32003, Taiwan; rkpanda277@gmail.com (P.K.P.); s1071127@mail.yzu.edu.tw (C.-H.L.); danny0935331003@gmail.com (Y.-X.T.)
[2] Department of Chemical Engineering, Massachusetts Institute of Technology, Cambridge, MA 02142, USA; ygandomi@mit.edu
* Correspondence: pcyang@saturn.yzu.edu.tw (P.-C.Y.); cthsieh@saturn.yzu.edu.tw (C.-T.H.)

Table S1. The K_{SV} and LOD parameters, together with the r^2 values, of both CND samples towards three metal ions.

Sample	CND			CND-100k		
	K_{sv} (ppm^{-1})	r^2	LOD (ppm)	K_{sv} (ppm^{-1})	r^2	LOD (ppm)
Fe^{3+}	0.2358	0.992	0.05	0.0443	0.997	0.28
Fe^{2+}	0.0226	0.999	0.56	0.0191	0.999	0.66
Hg^{2+}	0.0051	0.996	2.47	0.0078	0.999	1.62

Remark:

$$\sigma=\sqrt{\frac{1}{N}\sum_{i=1}^{N}(x_i-x)^2}$$

Table S2. Comparison among the LODs toward metal ions by different CND samples.

Method	detector/metal ion	LOD (ppm)	Reference
Hydrothermal synthesis	CND/Fe^{3+}	0.05	This work
Hydrothermal synthesis	CND/Fe^{2+}	0.56	This work
Hydrothermal synthesis	CND-100k/Hg^{2+}	1.62	This work
Liquid-phase synthesis	N-CQD/Hg^{2+}	2.4	[50]
Solvothermal synthesis	N-CQD/Hg^{2+}	1.0	[51]
Commercial product	Lysozyme/Fe^{3+}	0.1	[52]
Chemical reduction	Ag NP/Hg^{2+}	0.02	[53]
Chemical reduction	Au NP/Fe^{2+}	0.02	[28]

Remark: N-CQD: N-doped carbon quantum dot; Ag NPs: silver nanoplate; Au NPs: gold nanoparticle

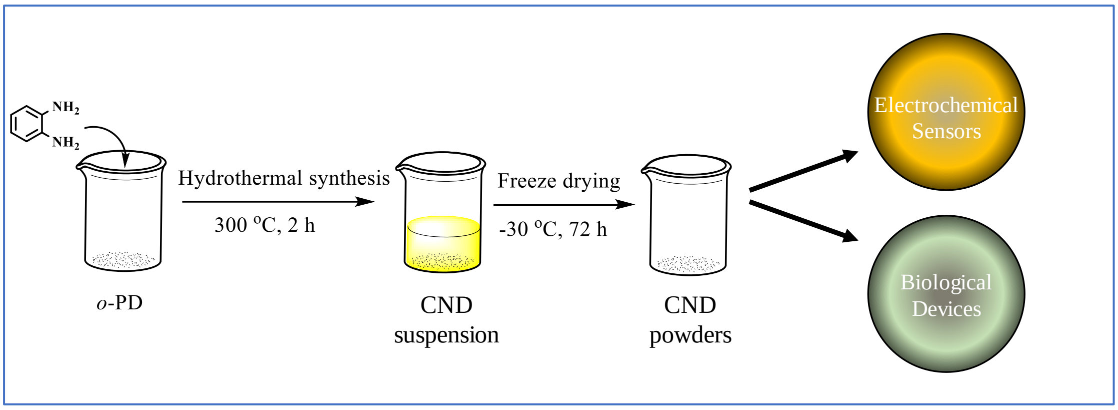

Figure S1. Schematic diagram for illustrating the preparation of CND powders and their application.

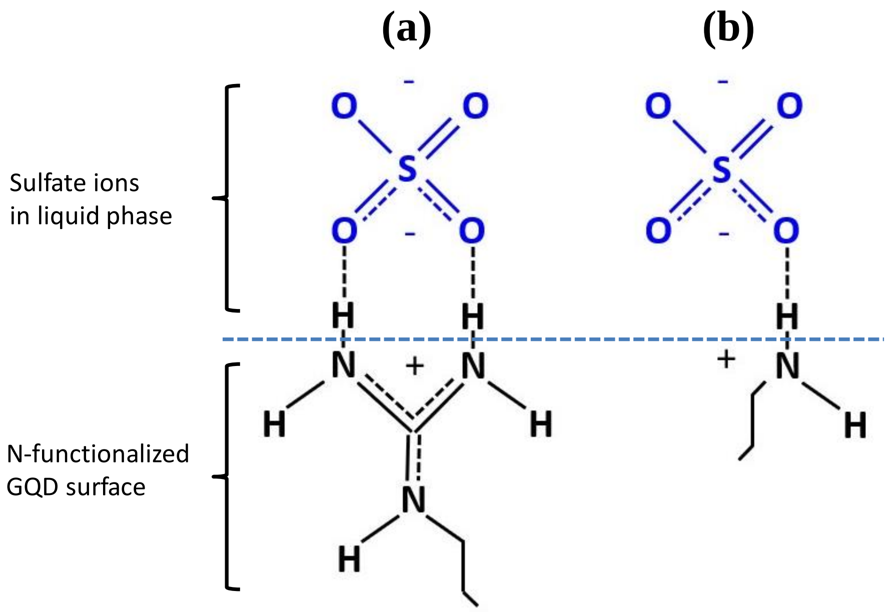

Figure S2. The interaction process through two pathways: (a) two neighboring amino groups via a bidentate hydrogen-bonding and (b) one single amino group via a monodentate hydrogen-bonding with hydrogen sulfate anions on N-functionalized CND surface.

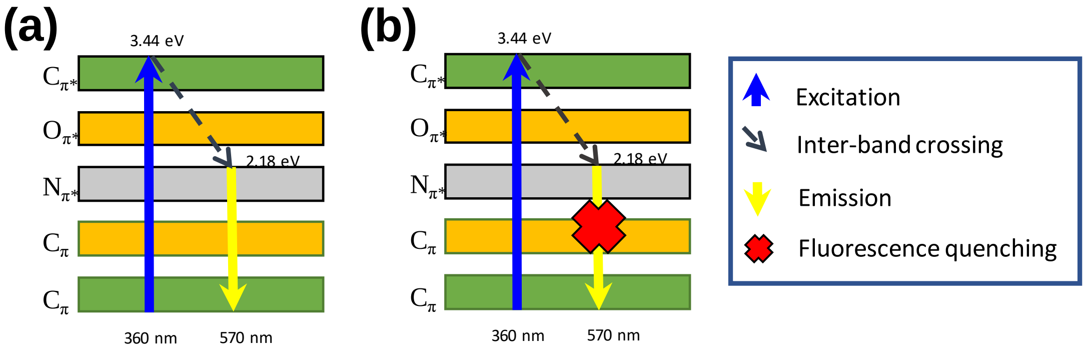

Figure S3. Inter-band gap structure models of CND sample under UV illumination: (a) before and (b) after detecting metal ions.
